# Supplementary material for: Seroprevalence of IgG Antibodies Directed against Dengue, Chikungunya and West Nile Viruses and Associated Risk Factors in Madagascar, 2011 to 2013
Source: Viruses. 2023 Aug 8;15(8):1707. doi: 10.3390/v15081707 (PMC10458928; doi:10.3390/v15081707)
Supplement: Supplementary file 1 [file viruses-15-01707-s001.zip › viruses-2544988-supplementary.pdf]

Supplementary Figure S1. Spatial distribution of seropositivity for the different DENV serotypes. (A) DENV1 serotype (B) DENV2 serotype (C) DENV3 serotype (D) DENV4 serotype.

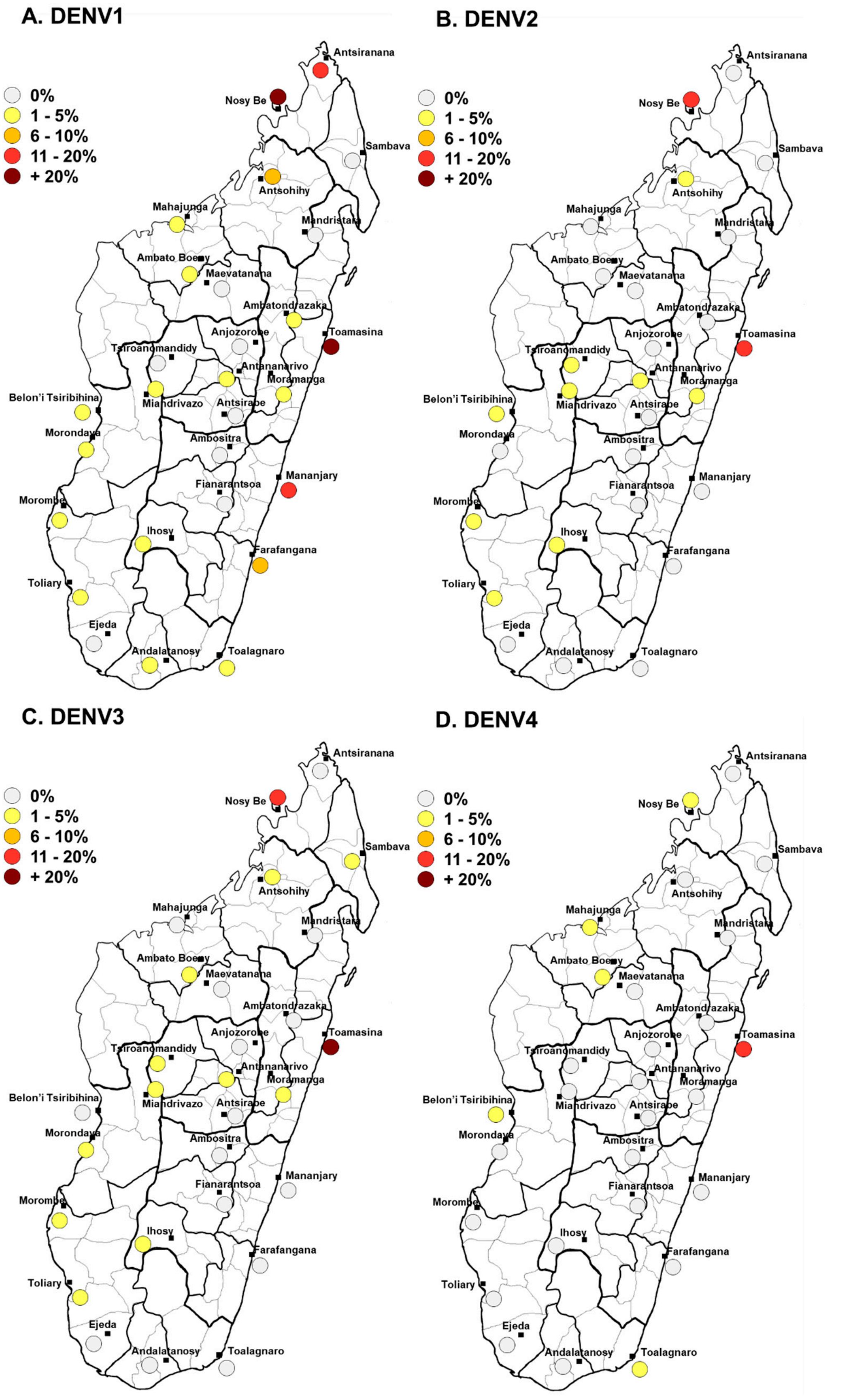

Supplementary Table S1. Univariate analysis results for Dengue virus seropositivity.

Logistic regression analysis was run using xtlogit command and variables with p-value less than or equal to 0.2 (in bold) were selected for multivariate analysis. Variable "General environment" was used in multivariate models over "Distance to SHC" as it was deemed to be more representative of the direct environment. Due to possible confusion bias and incoherent results, "Frequent use of bed net" variable was excluded from multivariate models.

Tested variable	N tested[1]	Nb pos (%)	IC pos	OR	IC95% OR	p-value
**Age category**						
18-24 years	388	26 (6.7)	3.5-9.9	1		
25-34 years	434	28 (6.5)	3.6-9.3	0.99	0.52-1.87	1.000
35-44 years	360	23 (6.4)	3.1-9.6	1.0	0.51-1.96	
≥45 years	498	33 (6.6)	4.0-9.3	0.99	0.53-1.85	
**Sex**						
Male	829	51 (6.2)	4.3-8.0	1		
Female	851	59 (6.9)	4.4-9.5	1.20	0.77-1.88	0.4188
**Distance from SHC**						
Close to SHC	840	45 (5.4)	3.1-7.6	1		
Distant from SHC	840	65 (7.7)	4.5-10.9	1.58	0.76-3.25	**0.2168**
**General environment**						
Rural	714	61 (8.5)	4.9-12.2	1		
Urban	516	28 (5.4)	2.3-8.6	0.54	0.23-1.24	0.2669
Peri-urban	450	21 (4.7)	2.5-6.9	0.58	0.25-1.35	
**MFA Factor 1**	1 680			1.17	0.86-1.61	0.3209
**MFA Factor 2**	1 680			1.87	1.36-2.59	**0.0001**
**MFA Factor 3**	1 680			1.17	0.77-1.77	0.4589
**MFA Factor 4**	1 680			2.19	1.45-3.30	**0.0002**
**Education level**						
Never been to school	214	14 (6.7)	3.1-10.2	1		
Primary school level	626	47 (7.5)	4.8-10.2	1.16	0.56-2.40	
Secondary school	500	34 (6.8)	4.1-9.5	1.12	0.51-2.45	0.5911
High school	260	12 (4.6)	1.9-7.3	0.77	29.6-1.99	
University	80	3 (3.8)	0.0-7.9	0.44	0.10-0.52	
**Outdoor profession**						
No	756	48 (6.3)	3.8-8.9	1		
Yes	924	62 (6.7)	4.3-9.1	1.13	0.67-1.93	0.6462
		**Smoker**				
No	1 336	93		1		
Yes	344	17		0.47	0.25-0.88	**0.0185**
**Frequent use of bed nets**						
No	466	18 (3.9)	1.6-6.1	1		
Yes	1 214	92 (7.6)	5.4-9.8	1.9	1.0-3.6	**0.0451**
		**Frequent work in rice fields**				
No	911	77 (8.5)	5.5-11.4	1		
Yes	769	33 (4.3)	2.6-6.0	0.41	0.23-0.72	**0.0021**
		**Frequent activities in the forest**				
No	1 084	58 (5.4)	3.4-7.3	1		
Yes	596	52 (8.7)	5.4-12.0	1.49	0.88-2.53	**0.1380**

**Frequent contacts to water bodies (fishing, swimming)**						
No	1 564	107 (6.8)	4.8-8.9	1		
Yes	116	3 (2.6)	0.0-5.4	0.57	0.16-2.08	0.3933
**Electricity in the house**						
No	1 114	75 (6.7)	4.2-9.2	1		
Yes	564	35 (6.2)	3.1-9.3	0.86	0.44-1.67	0.6596
**Construction materials of the house**						
"Hard" materials (bricks, wood, tin)	1 085	61 (5.6)	3.8-7.5	1		
Contains "soft" materials (cob or vegetable house)	593	49 (8.3)	4.9-11.6	1.58	0.91-2.76	**0.1038**
**Running water in the house**						
No	1 593	108 (6.8)	4.7-8.8	1		
Yes	87	2 (2.3)	0.0-5.1	0.28	0.05-1.64	**0.1589**
**Smoke in the house when cooking**						
No	1 110	62 (5.5)	3.7-7.5	1		
Yes	568	48 (8.5)	5.1-11.8	1.70	1.02-2.83	**0.0400**
**House targeted for Indoor Residual Spraying program (last 12 months)**						
No	1 190	103 (8.7)	6.1-11.3	1		
Yes	488	7 (1.4)	0.3-2.6	0.17	0.07-0.44	**0.0003**
**Biological wastes next to the house**						
No	279	15 (5.4)	2.2-8.5	1		
Yes	1 399	95 (6.8)	4.6-9.0	1.02	0.49-2.09	0.9665
**Non-biological wastes next to the house**						
No	457	18 (3.9)	2.0-5.9	1		
Yes	1 221	92 (7.5)	5.0-10.0	1.56	0.82-2.94	**0.1732**
**Cultures in the 10m around the house**						
No	736	31 (4.2)	2.7-5.7	1		
Yes	942	79 (8.4)	5.3-11.5	1.64	0.93-2.90	**0.0900**
**Water in the 10m around the house**						
No	1 436	90 (6.3)	4.3-8.3	1		
Yes	242	20 (8.3)	2.6-13.9	1.65	0.80-3.41	**0.1740**

Supplementary Table S2. Univariate analysis results for Chikungunya virus seropositivity.

Logistic regression analysis was run using xtlogit command and variables with p-value less than or equal to 0.2 (in bold) were selected for multivariate analysis. Variable "General environment" was used in multivariate models over "Distance to SHC" as it was deemed to be more representative of the direct environment. Due to possible confusion bias and incoherent results, "Frequent use of bed net" variable was excluded from multivariate models.

Tested variable	N tested[1]	Nb pos (%)	IC pos	OR	IC95% OR	p-value
**Age category**						
18-24 years	388	52 (13.4)	8.4-18.4	1		0.8143
25-34 years	434	56 (12.9)	7.6-18.2	1.08	0.56-2.08	
35-44 years	360	56 (15.6)	9.9-21.2	1.30	0.66-2.54	
≥45 years	498	66 (13.3)	8.3-18.2	0.96	0.51-1.82	
**Sex**						
Male	829	109 (13.1)	8.7-17.6	1		
Female	851	121 (14.2)	9.8-18.6	0.99	0.63-1.57	0.9679
**Distance from SHC**						
Close to SHC	840	124 (14.8)	9.8-19.7	1		
Distant from SHC	840	106 (12.6)	5.9-19.3	0.45	0.14-1.44	**0.1767**
**General environment**						
Rural	714	104		1		**0.0158**
Urban	450	21		2.69	0.78-9.32	
Peri-urban	516	105		0.39	0.09-1.62	
**MFA Factor 1**	1 680			0.92	0.55-1.54	0.7531
**MFA Factor 2**	1 680			2.80	1.52-5.16	**0.0009**
**MFA Factor 3**	1 680			0.51	0.26-1.01	**0.0541**
**MFA Factor 4**	1 680			2.29	1.06-4.93	**0.0343**
**Education level**						
Never been to school	214	26 (12.1)	4.2-20.1	1		0.8993
Primary school level	626	80 (12.8)	7.5-18.1	0.81	0.37-1.78	
Secondary school	500	81 (16.2)	10.8-21.6	1.01	0.43-2.42	
High school	260	27 (10.4)	6.0-14.8	0.75	0.28-2.02	
University	80	16 (20.0)	10.7-29.3	0.96	0.29-3.16	
**Outdoor profession**						
No	756	134 (17.7)	12.6-22.8	1		
Yes	924	96 (10.4)	5.5-15.3	0.55	0.28-1.05	**0.0711**
**Smoker**						
No	1 336	179 (13.4)	9.2-17.6	1		
Yes	344	51 (14.8)	9.2-20.5	1.48	0.84-2.61	**0.1725**
**Frequent use of bed nets**						
No	466	34 (7.3)	3.8-10.8	1		
Yes	1 214	196 (16.1)	11.3-21.0	1.84	0.95-3.56	**0.0689**
**Frequent work in rice fields**						
No	911	162 (17.8)	12.8-22.7	1		
Yes	769	68 (8.8)	3.6-14.1	0.47	0.24-0.94	**0.0312**

**Frequent activities in the forest**						
No	1 084	133 (12.3)	8.5-16.1	1		
Yes	596	97 (16.3)	8.9-23.7	1.80	0.96-3.40	**0.0689**
**Frequent contacts to water bodies (fishing, swimming)**						
No	1 564	208 (13.3)	9.5-17.1	1		
Yes	116	22 (19.0)	0.0-41.8	1.71	0.47-6.25	0.4162
**Electricity in the house**						
No	1 114	122 (11.0)	5.8-16.1	1		
Yes	564	108 (19.1)	12.8-25.5	1.19	0.56-2.55	0.6452
**Construction materials of the house**						
"Hard" materials (bricks, wood, tin)	1 085	148 (13.6)	9.4-17.9	1		
Contains "soft" materials (cob or vegetable house)	593	82 (13.8)	7.9-19.7	0.61	0.32-1.18	**0.1432**
**Running water in the house**						
No	1 593	218 (13.7)	9.4-18.0	1		
Yes	87	12 (13.8)	3.5-24.1	1.31	0.35-4.83	0.6896
**Smoke in the house when cooking**						
No	1 110	168 (15.1)	10.4-19.8	1		
Yes	568	62 (10.9)	6.0-15.8	1.05	0.58-1.89	0.8818
**House targeted for anti-mosquito spraying program (last 12 months)**						
No	1 190	228 (19.2)	13.7-24.6	1		
Yes	488	2 (0.4)	0.0-1.0	0.02	0.00-0.23	**0.0026**
**Biological wastes next to the house**						
No	279	34 (12.2)	5.6-18.8	1		
Yes	1 399	196 (14.0)	9.4-18.7	1.29	0.63-2.65	0.4877
**Non-biological wastes next to the house**						
No	457	25 (5.5)	2.8-8.1	1		
Yes	1 221	205 (16.8)	11.6-21.9	1.95	0.99-3.84	**0.0530**
**Cultures in the 10m around the house**						
No	736	65 (8.8)	5.1-12.6	1		
Yes	942	165 (17.5)	11.4-23.6	1.07	0.59-1.96	0.8183
**Water in the 10m around the house**						
No	1 436	182 (12.7)	8.4-17.0	1		
Yes	242	48 (19.8)	10.8-28.9	1.58	0.75-3.33	0.2288

Supplementary Table S3. Univariate analysis results for West Nile virus seropositivity.

Logistic regression analysis was run using xtlogit command and variables with p-value less than or equal to 0.2 (in bold) were selected for multivariate analysis. Variable "General environment" was used in multivariate models over "Distance to SHC" as it was deemed to be more representative of the direct environment. Due to possible confusion bias and incoherent results, "Frequent use of bed net" variable was excluded from multivariate models.

Tested variable	N tested[1]	Nb pos (%)	IC pos	OR	IC95% OR	p-value
**Age category**						
18-24 years	388	47 (12.1)	8.5-15.8	1		
25-34 years	434	48 (11.3)	8.1-14.5	0.83	0.51-1.35	0.2498
35-44 years	360	51 (14.2)	10.1-18.2	1.33	0.81-2.17	
≥45 years	498	67 13.5)	9.8-17.1	1.17	0.74-1.86	
**Sex**						
Male	829	106 (12.8)	10.0-15.6	1		
Female	851	108 (12.7)	9.8-15.5	1.06	0.76-1.46	0.7432
**Distance from SHC**						
Close to SHC	840	71 (8.5)	6.3-10.6	1		
Distant from SHC	840	143 (17.0)	12.8-21.2	2.48	1.49-4.13	**0.0005**
**General environment**						
Rural	714	118 (16.5)	11.9-21.1	1		
Urban	450	59 (7.2)	4.5-9.9	0.37	0.20-0.68	**0.0052**
Peri-urban	516	37 (13.1)	9.0-17.2	0.79	0.44-1.42	
**MFA Factor 1**	1 680			1.07	0.84-1.36	0.5736
**MFA Factor 2**	1 680			1.48	1.15-1.90	**0.0020**
**MFA Factor 3**	1 680			1.62	1.20-2.17	**0.0014**
**MFA Factor 4**	1 680			1.09	0.77-1.55	0.6127
**Education level**						
Never been to school	214	40 (19.0)	13.1-25.0	1		
Primary school level	626	103 (16.5)	12.4-20.6	0.96	0.60-1.54	
Secondary school	500	48 (9.6)	6.7-12.5	0.56	0.33-0.96	**0.0051**
High school	260	22 (8.5)	4.9-12.0	0.52	0.27-1.00	
University	80	1 (1.3)	0.0-3.7	0.07	0.01-52.5	
**Outdoor profession**						
No	756	69 (9.1)	6.4-11.8	1		
Yes	924	145 (15.7)	12.4-19.0	1.87	1.26-2.77	**0.0019**
		**Smoker**				
No	1 336	171 (12.8)	10.2-15.4	1		
Yes	344	43 (12.5)	8.8-16.2	0.93	0.62-1.40	0.7214
**Frequent use of bed nets**						
No	466	32 (6.9)	4.1-9.6	1		
Yes	1 214	182 (15.0)	12.0-18.0	1.86	1.17-2.96	**0.0085**
**Frequent work in rice fields**						
No	911	95 (10.4)	7.8-13.0	1		
Yes	769	119 (15.5)	11.8-19.2	1.57	1.05-2.34	**0.0268**

**Frequent activities in the forest**						
No	1 084	108 (10.0)	7.8-12.1	1		
Yes	596	106 (17.8)	13.4-22.5	1.64	1.13-2.37	**0.0089**
**Frequent contacts to water bodies (fishing, swimming)**						
No	1 564	195 (12.5)	10.0-14.9	1		
Yes	116	19 (16.4)	8.1-24.6	2.12	1.10-4.09	**0.0242**
**Electricity in the house**						
No	1 114	180 (16.2)	12.8-19.5	1		
Yes	564	34 (6.0)	3.7-8.3	0.33	0.20-0.55	**0.0000**
**Construction materials of the house**						
"Hard" materials (bricks, wood, tin)	1 085	97 (8.9)	6.9-10.9	1		
Contains "soft" materials (cob or vegetable house)	593	117 (19.7)	15.1-24.4	2.03	1.37-3.02	**0.0005**
**Running water in the house**						
No	1 593	210 (13.2)	10.6-15.7	1		
Yes	87	4 (4.6)	0.6-8.6	0.43	12.3-1.49	**0.1822**
**Smoke in the house when cooking**						
No	1 110	140 (12.6)	9.7-15.5	1		
Yes	568	74 (13.0)	9.5-16.5	0.99	0.61-1.62	0.4808
**House targeted for anti-mosquito spraying program (last 12 months)**						
No	1 190	156 (13.1)	103-15.9	1		
Yes	488	58 (11.9)	7.7-16.1	0.73	0.44-1.21	0.2194
**Biological wastes next to the house**						
No	279	25 (9.0)	4.9-13.0	1		
Yes	1 399	189 (13.5)	10.8-16.2	1.29	0.74-2.24	0.3634
**Non-biological wastes next to the house**						
No	457	52 (11.3)	7.4-15.3	1		
Yes	1 221	162 (13.3)	10.5-16.1	0.92	0.60-1.42	0.7118
**Cultures in the 10m around the house**						
No	736	62 (8.4)	6.1-10.8	1		
Yes	942	152 (16.1)	12.6-19.7	1.62	1.06-2.46	**0.0252**
**Water in the 10m around the house**						
No	1 436	187 (13.0)	10.4-15.6	1		
Yes	242	27 (11.1)	6.5-15.9	1.00	0.55-1.82	0.9873
